# Transesophageal echocardiography-guided percutaneous patent ductus arteriosus closure without fluoroscopy

**DOI:** 10.1186/s13019-023-02248-8

**Published:** 2023-04-15

**Authors:** Gang Wang, Yuhao Wu, Zhengxia Pan, Chun Wu, Yonggang Li, Hongbo Li, Quan Wang, Bo Liu, Jiangtao Dai

**Affiliations:** 1 Department of Cardiothoracic Surgery, Children’s Hospital of Chongqing Medical University, Chongqing, China; 2 Ministry of Education Key Laboratory of Child Development and Disorders, National Clinical Research Center for Child Health and Disorders, Chongqing Key Laboratory of Pediatrics, China International Science and Technology Cooperation Base of Child Development and Critical Disorders, Chongqing, China

**Keywords:** Transesophageal echocardiography, Patent ductus arteriosus, Femoral vein, Percutaneous closure

## Abstract

**Objectives:**

A retrospective study was performed to summarize the experience of transcatheter closure of patent ductus arteriosus (PDA) through the right femoral vein under the guidance of transesophageal echocardiography (TEE).

**Methods:**

From January 2019 to September 2021, 75 children who underwent PDA closure through the right femoral vein under the guidance of TEE were included. The guide wire and delivery sheath were inserted through the ductus arteriosus into the descending aorta via the right femoral vein, and the occluder was subsequently deployed. After discharge, all patients were required for outpatient follow-ups at 1, 3, 6 and 12 months.

**Results:**

In this group, patients were older than 10 months of age and body weight greater than 8 kg. Among 75 cases with PDA, 63 were tubular type and 12 were conical type. The mean operative time was 40.2 ± 7.3 min. The size of PDA occluder ranged from 4–6 to 12–14 mm. The mean hospital stay was 5.5 ± 0.5 days. One month after discharge, there were 4 cases with a mild residual shunt. Eventually, the residual shunt was not observed during 3, 6, and 12 months of follow-up.

**Conclusions:**

PDA closure under the guidance of TEE can be performed through the right femoral vein successfully and effectively. This procedure has no contrast agent usage, radiation exposure, or open incisions.

## Introduction

The ductus arteriosus is a normal fetal artery connecting the aorta and the pulmonary artery. Patent ductus arteriosus (PDA) is a series of pathophysiological changes caused by the blood diversion from the aorta to the pulmonary artery due to the delayed closure of the ductus arteriosus in children. The incidence of PDA in infants is estimated to be 57 per 100,000 live births in children [1]. Leaving untreated, heart failure and pulmonary hypertension cannot be avoided at the end stage. The main treatments for PDA in term infants include open surgery, percutaneous closure under fluoroscopy guidance, thoracoscopic PDA closure [2], transthoracic PDA closure [3–5]. Although percutaneous closure under fluoroscopy has become the preferred therapeutic option, potential radiation exposure still cannot be ignored for physicians and patients. Using open surgery, thoracoscopy, or transthoracic PDA closure, surgical incisions and complications such as pulmonary collapse, pneumothorax, and pleural effusion cannot be avoided. PDA closure through the right femoral vein under the guidance of TEE is a safe and effective surgical approach [6, 7]. However, reports regarding this technique are scarce. Herein, we report PDA closure through the right femoral vein under the guidance of TEE in 75 children.

## Methods

### Study design and data collection

This retrospective study was approved by the Institutional Ethics Committee of Children’s Hospital of Chongqing Medical University. Informed consent was obtained from the parents. This study complied with the declaration of Helsinki. One attending doctor and two nurses were in charge of the data collection. Data were obtained from the institutional database. This database included basic characteristics of patients, perioperative examinations, surgical records, and postoperative complications. Follow-up data were collected from the outpatient medical system. Therefore, missing data regarding perioperative information were less likely. All patients were required for outpatient follow-up at 1, 3, 6 and 12 months after the initial operation. Our follow-up nurses maintained close contact with the patients via telephone or social media.

### Study population

In total, 75 children who underwent PDA closure through the right femoral vein under the guidance of TEE at Children’s Hospital of Chongqing Medical University from January 2019 to September 2021 were consecutively included. Patients who met the following criteria were included: (1) Diagnosis of PDA confirmed by preoperative echocardiography; (2) Patients older than 10 months of age and body weight greater than 8 kg; (3) The size of PDA ranged from 3 to 8 mm; (4) Patients with combined heart failure, elevated pulmonary artery pressure, or recurrent pulmonary infection. Patients who met the following criteria were excluded: (1) Patients with infective endocarditis or right-to-left shunt due to pulmonary hypertension; (2) Patients in combination with other cardiac defects requiring open surgery; (3) Patients with PDA-dependent heart defects. All children underwent physical examinations, routine blood tests, echocardiography, chest x-ray, and ECG before procedure.

### Device and surgical procedure

**Fig. 1:**
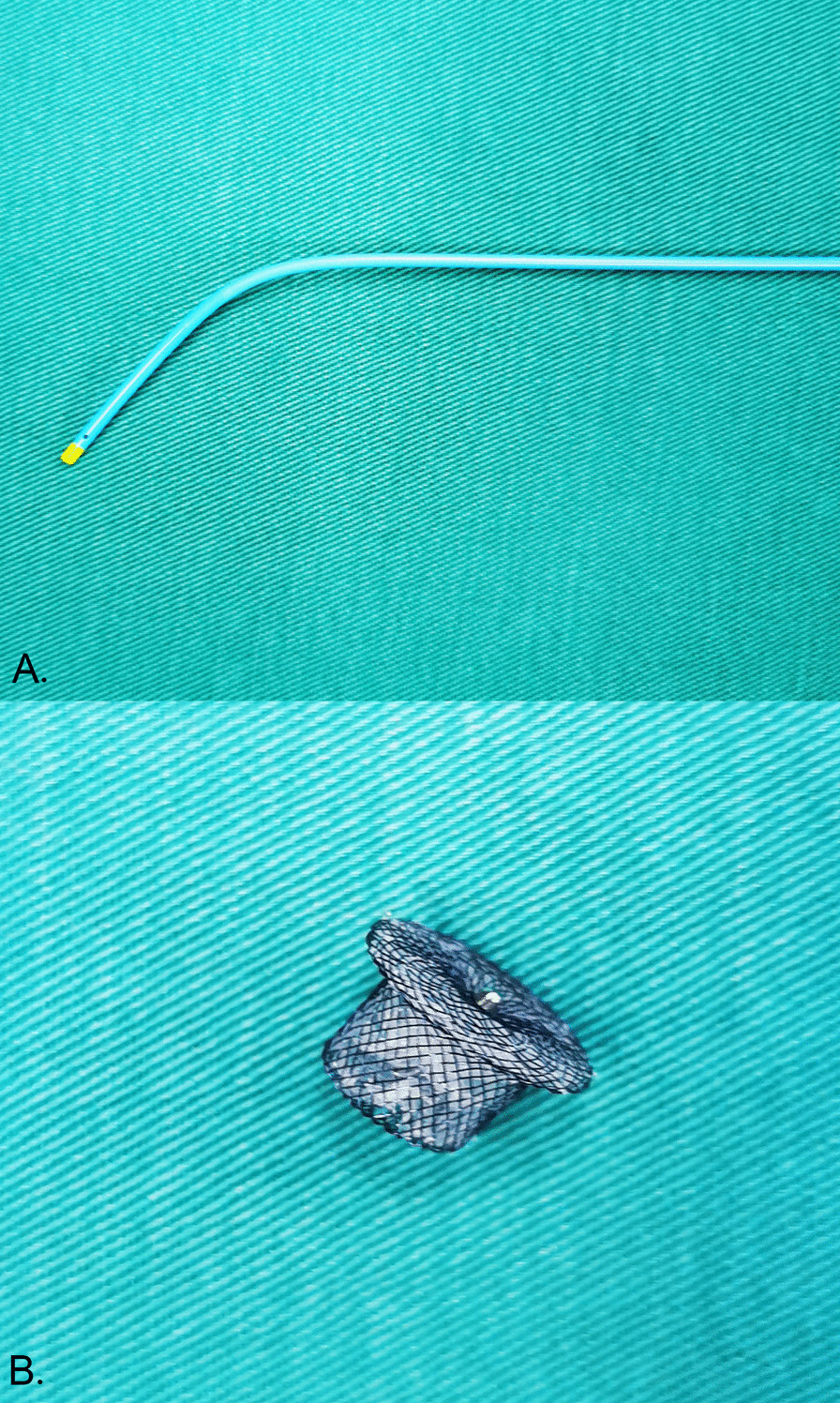
Nickel–titanium–alloy PDA occluder and delivery system. **A** The 5F catheter; **B** Nickel–titanium–alloy PDA occluder

Nickel-titanium-alloy PDA occluder and delivery system (PDA occluder, Shape Memory Alloy Co., Ltd, Shanghai), which is composed of a 5F catheter (Fig. 1A), a delivery sheath, a delivery guide wire, and an occluder (Fig. 1B), were used for using interventional closure.

**Fig. 2:**
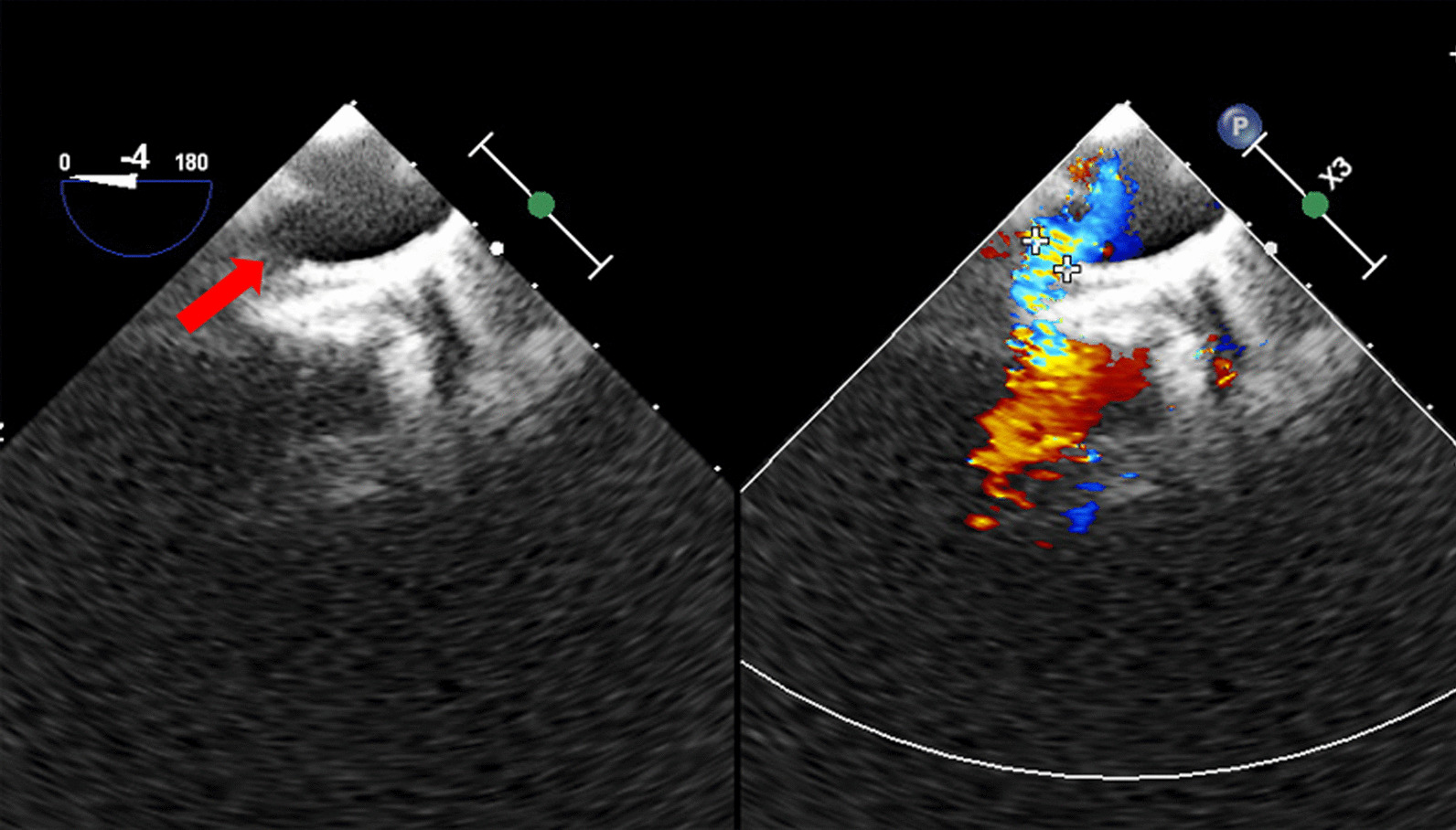
Measurements of the PDA diameter. (Red arrow indicates the ductus arteriosus)

**Fig. 3:**
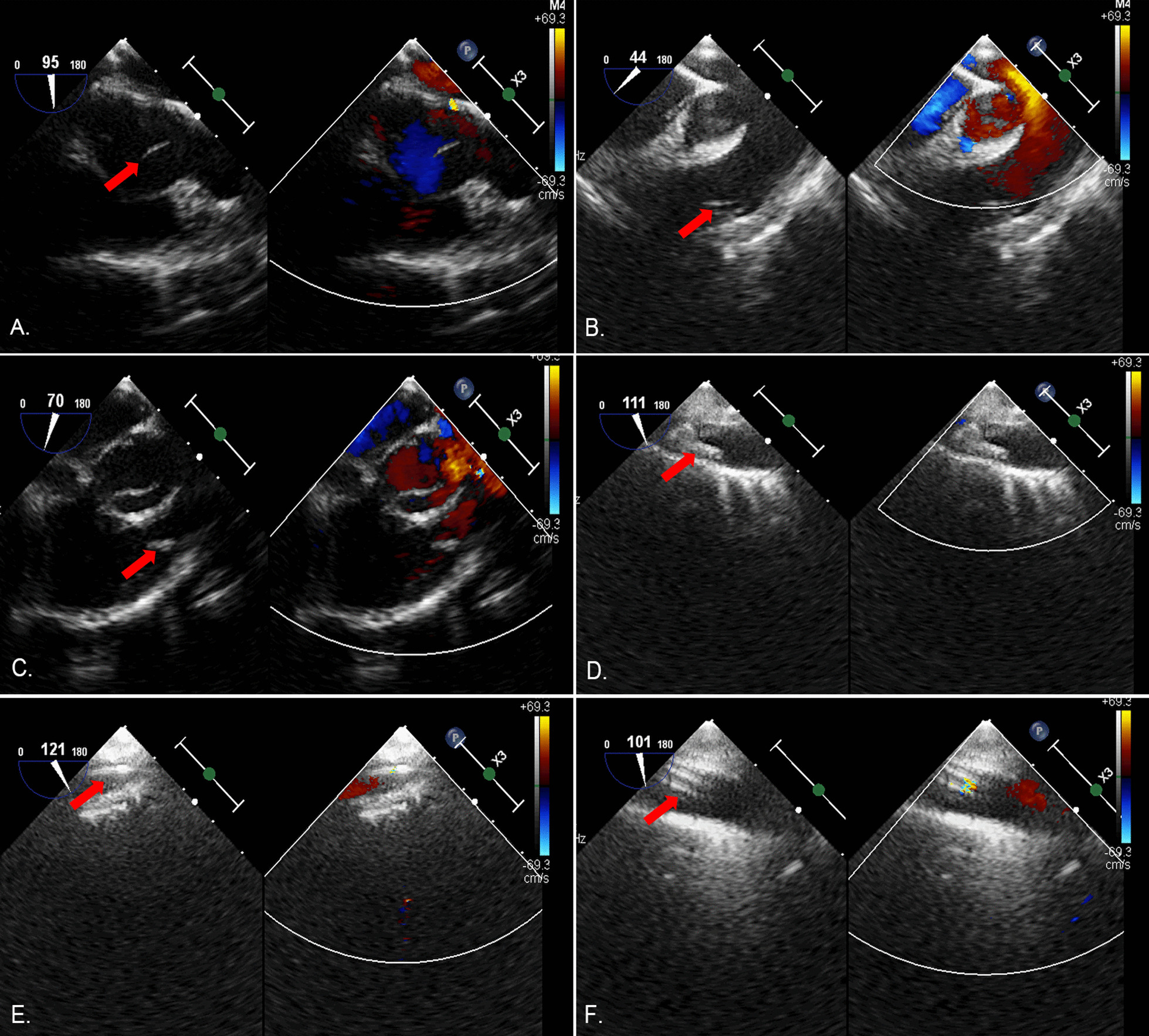
Perioperative procedures of PDA closure under the guidance of TEE. **A** Red arrow indicates the 5F catheter facing to the tricuspid valve; **B** red arrow indicates the guide wire entering the right ventricular outflow tract; **C** red arrow indicates the guide wire entering the pulmonary artery; **D** red arrow indicates the guide wire entering the ductus arteriosus; **E** red arrow indicates the guide wire entering the descending aorta; **F** red arrow indicates the delivery sheath entering the ductus arteriosus

**Fig. 4:**
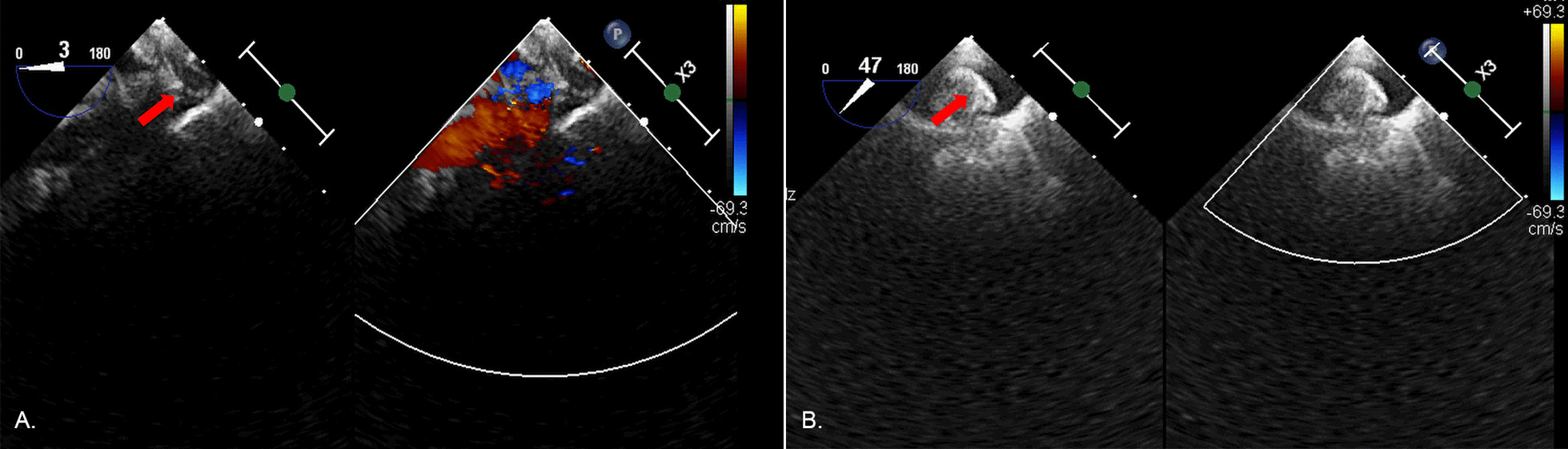
Occluder deployment under the guidance of TEE. **A** Red arrow indicates the deployment of the aortic side disc; **B** red arrow indicates the deployment of the pulmonary side disc

A total of 4 surgeons performed this procedure, and a surgical team was readily available in case of device migration. After successful general anesthesia, upper and lower limp blood pressure was obtained and monitored. Length and diameters of the ductus arteriosus were measured using TEE (Fig. 2). The occluder size was selected according to the TEE results and was generally 3–4 mm larger than the diameter of the ductus arteriosus. During this procedure, the patient was administered with a dose of 1 mg/kg heparin. Percutaneous puncture of the right femoral vein was conducted, and a venous sheath was placed. A guide wire and a 5F catheter were successively inserted through the sheath. The TEE probe was adjusted to 90° and 125° to ensure that the guide wire and the 5F catheter could enter the inferior vena cava and the right atrium. Then, we rotated the 5F catheter so that the tip could face the tricuspid valve (Fig. 3A). When the TEE probe was adjusted to 60 degrees, the guide wire and the 5F catheter were inserted into the right ventricle. The 5F catheter was rotated again to facilitate the guide wire entering the right ventricular outflow tract (Fig. 3B) and pulmonary artery (Fig. 3C). The TEE probe was adjusted to 125 degrees displaying the ductus arteriosus, and the guide wire was advanced via the ductus arteriosus to guide the 5F catheter into the descending aorta (Fig. 3D, E). We double checked that images were correctly labeled. The 5F catheter was replaced by a delivery sheath which was also inserted into the descending aorta via the ductus arteriosus (Fig. 3F). The guide wire and the inner core of the delivery sheath were extracted, and an occluder was slowly inserted along the delivery sheath (Fig. 4A, B). Under TEE guidance, the aortic side of occluder was released first. Then, the delivery sheath was withdrawn and the pulmonary side of occluder was released. Ultimately, the push-and-pull test was performed to make sure that the occluder was deployed stably.

### Post-operative treatment and follow-up

Following closure, heparin was neutralized with the use of protamine. Ventilation was ceased after the patient woke up. For anticoagulant therapy, patients were given oral aspirin for 3 months postoperatively. Each patient was required for echocardiography during the follow-up at 1, 3, 6 and 12 months after discharge.

### Statistical analysis

Descriptive data were presented as the means ± standard deviation as appropriate.

## Results

The average age of included patients was 2.9 ± 1.7 years, with a range of 10 months to 12.7 years. The average body weight was 10.2 ± 6.7 kg, with a range of 8–35 kg. Among the 75 cases, 63 cases were tubular type and 12 cases were conical type. The average diameter of ductus arteriosus was 6.5 ± 2.6 mm, with a range of 3–8 mm. The average procedure time was 40.2 ± 7.3 min, with a range of 20 min to 2 h. The range of PDA occluder size was from 4–6 to 12–14 mm (Table 1).

**Table 1:** Patients’ characteristics

Patients’ characteristics	
Gender, n, male/female	33/42
Surgical age, years, mean ± SD	2.95 ± 1.75
Weight, kg, mean ± SD	10.25 ± 6.75
Type of PDA, n, tubular/conical	63/12
Diameters of ductus arteriosus, mm, mean ± SD	6.5 ± 2.6
Procedure time, minutes, mean ± SD	40.2 ± 7.3
Size of occluder, mm, range*	4–6 to 12–14
Hospital stay, days, mean ± SD	5.5 ± 0.5

*SD* standard deviation

*4/12 mm represents the pulmonary side disc of the occluder, and 6/14 mm represents the aortic side disc of the occluder

**Fig. 5:**
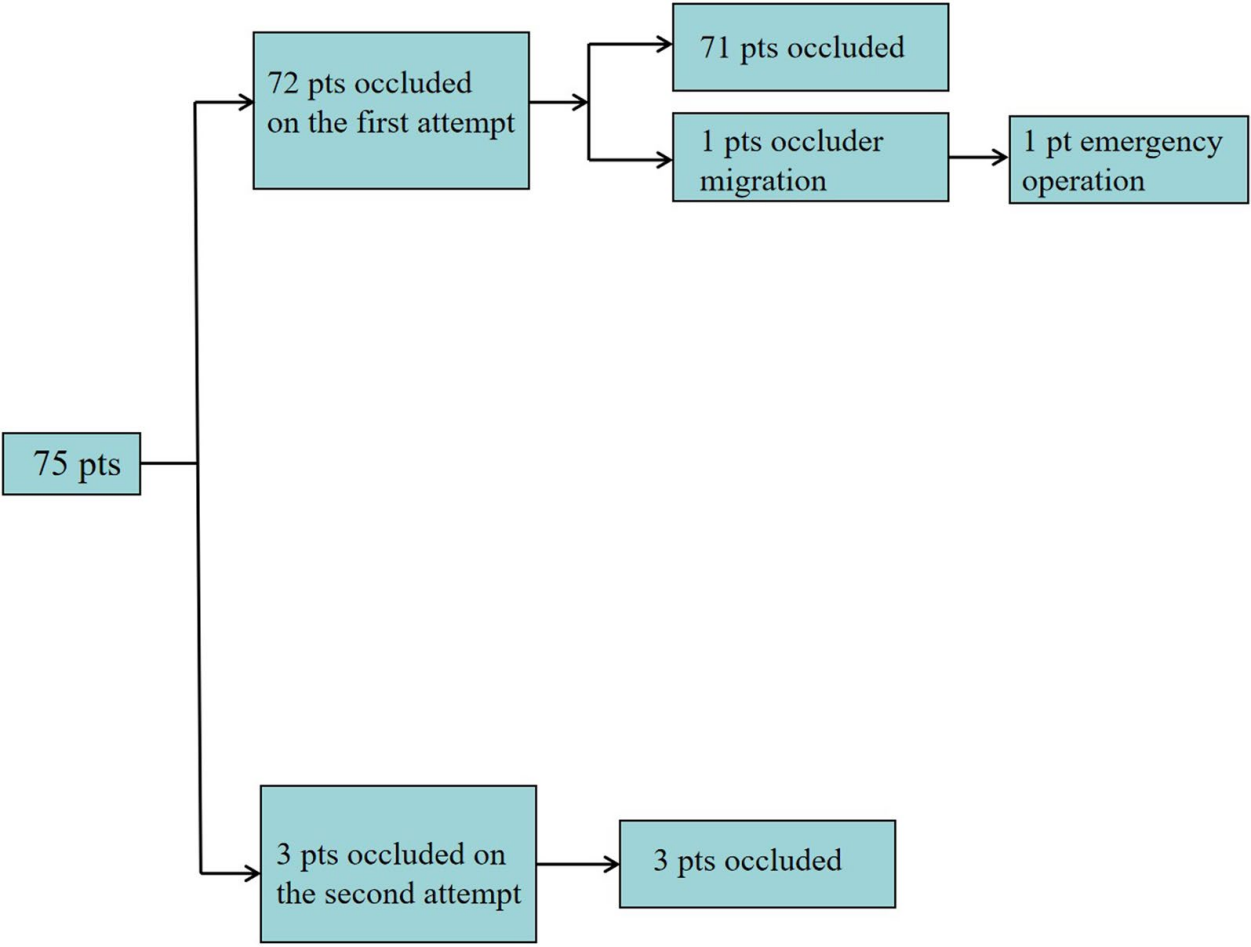
Outcome schema of the entire cohort. A total of 74 patients are successfully occluded. Occluder migration is noted in one patient, and an emergency thoracotomy is performed to remove the occluder. 71 patients are successfully occluded with selected occluder. In 3 patients with PDA, a secondary attempt is required using a larger size of occluder

A total of 74 cases were successfully occluded, and the average length of hospital stay was 5.5 ± 0.5 days. Occluder migration was noted in only one case. An emergency thoracotomy was performed to remove the occluder, and the ductus arteriosus was finally sutured. 71 PDA cases were successfully occluded with selected occluder. In 3 PDA cases, a secondary attempt was required using a larger size of occluder (Fig. 5). However, occluder migration was observed in one case due to patient-device size mismatch. An emergency thoracotomy was then performed to remove the occluder, and the ductus arteriosus was sutured. Combined atrial septal defects were also occluded simultaneously in three children.

Under TEE monitoring, there were no residual shunt, valvular regurgitation, or aortic and pulmonary artery obstruction after closure. There was no case with a residual gradient across the aortic arch. All patients underwent 1 year outpatient follow-up. There were 4 cases with mild residual shunt 1 month after discharge based on transthoracic echocardiography. The residual shunts were hemodynamically insignificant, and no medical treatment was required. Eventually, residual shunt was resolved at 3- to 6-month follow-up. There were no complications such as pericardial effusion, recurrent laryngeal nerve injury, hemolysis, thrombosis or infective endocarditis during the follow-up.

## Discussion

Currently, the main approaches for PDA treatment in infants and children are open surgery, percutaneous closure under fluoroscopy guidance, thoracoscopic PDA closure, and transthoracic PDA closure via TTE guidance. Open surgery generally has a large surgical incision affecting the appearance and chest development in children. Porstmann et al. [8] was the first to consider using percutaneous interventional therapy and achieved success in 1967. So far, closure under fluoroscopy guidance has been widely performed [9, 10]. However, fluoroscopic guidance exposes physicians and children under radiation directly. Additionally, children may be prone to allergic to contrast agents. Thoracoscopic PDA closure has relatively high risks including bleeding and recurrent laryngeal nerve injury [2, 11]. In recent years, PDA closure through small thoracic incision under TEE guidance has been gradually reported [5]. Although radiation exposure and contrast agents are avoided using this technique, there is still a surgical incision left on the chest.

Whether PDA can be minimally occluded through femoral vein under the guidance of TEE remain debatable. The feasibility of this method has been proved after successful animal experiments through veins under the guidance of TEE [12, 13]. At present, TEE-guided transcatheter femoral vein occlusion of PDA has achieved certain clinical outcomes. This method successfully occludes PDA through femoral vein without using contrast agents and X-ray. In addition, there are no severe complications indicating the safety and effectiveness of this technique [14]. However, this technique also has some limitations. First, TEE guidance needs critically technical requirements. Compared with fluoroscopy guidance which is easy to determine the location of the catheter as fluoroscopy detects through projection, echocardiography detects by facets and cannot show the position of the catheter accurately. Second, TEE-guided percutaneous intervention can lead to significantly technical difficulties and a lengthy learning curve. Without fluoroscopy guidance, the spatial perception of the surgeon and timely communication with the sonographer are particularly important.

To the best of our knowledge, this is the largest cohort which explores the safety and effectiveness of transcatheter femoral vein occlusion of PDA under TEE guidance in children. In our center, the longest surgical time at the initial stage was 2 h, and the shortest time was 20 min with an average duration of 40.2 min, which indicates that this method represents a better alternative than other methods considering the learning curve [14]. However, it is also noteworthy that cases with surgical time longer than 2 h possibly need to be changed to open surgery to avoid the increased risks due to the prolonged operation. Learning curve of this procedure, clinical acuity and ductus arteriosus size contribute to prolonged duration of this procedure. The results of this study showed that 71 cases were successfully occluded on the first attempt, but occluder migration was observed in one case. An emergency thoracotomy was then performed to remove the occluder, and the ductus arteriosus was sutured. Other 3 cases were occluded with a larger size occluder on the second attempt. Four cases with mild residual shunt were found during the follow-up 1 month after discharge. However, the residual shunt was resolved 3- to 6-month follow-up after discharge. We speculate that the residual shunt may be cured after the surface of the occluder disc is covered by endothelial cells [15].

The key procedure of this technique is that surgeons and sonographers cooperate closely at two angles of rotation to insert the catheter into the pulmonary artery. The first angle of rotation makes the tip of the 5F catheter face the tricuspid valve after entering the right atrium. The second angle of rotation makes the tip face the right ventricular outflow tract and pulmonary artery. After the guide wire enters the descending aorta, the guide wire must be further inserted nearly 20 cm to prevent the guide wire from sliding out. Surgeons insert the delivery sheath to the descending aorta, release the occluder disc under TEE guidance, and withdraw the delivery sheath to make the disc occlude the ductus arteriosus. The whole process need to be completed cautiously under the guidance of TEE. Occluder migration is a severe complication, therefore, operators need to observe the position of the occluder closely, check the location and shape of the occluder before deployment, and pay close attention to whether the location of the disc tail at the pulmonary side is fully visible. The location and shape of the occluder are also the key indicators during the follow-up.

Our experiences of transvenous PDA occlusion are acknowledged as follows: (1) surgeons should perform this procedure on appropriate patients, and fit size of occluder should be selected based on the TEE results; (2) surgeons and sonographers with sufficient knowledge of cardiac anatomy and echocardiography are required. Especially when entering the tricuspid valve, right ventricular outflow tract and pulmonary artery, they need to cooperate, calibrate and adjust the guide wire and catheter at two angles of rotation; (3) surgeons should mark the working distance and locations of the catheter in vitro before operation, which is convenient to find the path when the catheter need to be placed repeatedly; (4) when the delivery sheath cannot be inserted into the ductus arteriosus, it often indicates a distorted ductus arteriosus. To avoid rupture of the ductus arteriosus, repeated attempts are not recommended, and open surgery should be considered; (5) the diameter of the descending aorta, left pulmonary artery and blood pressure of upper and lower limbs need to be measured before and after operation to avoid obstruction of the aortic arch and left pulmonary artery after occluder deployment.

## Conclusions

In conclusion, transvenous PDA occlusion in children under the guidance of TEE is a safe and effective technique. This technique can avoid surgical incisions and radiation exposure to children and physicians. Further large-scale cohort studies need to be performed to determine the effectiveness and safety of this technique in younger and smaller patients.

## Data Availability

The data supporting the conclusions of this article will be made available by the authors upon request.
